# Deletion of the Mitochondrial Membrane Protein Fam210b Is Associated with the Development of Systemic Lupus Erythematosus

**DOI:** 10.3390/ijms25137253

**Published:** 2024-07-01

**Authors:** Yaqi Xu, Ran Gao, Min Zhang, Qi Zeng, Gaizhi Zhu, Jinming Qiu, Wenting Su, Renxi Wang

**Affiliations:** 1 Beijing Institute of Brain Disorders, Laboratory of Brain Disorders, Ministry of Science and Technology, Collaborative Innovation Center for Brain Disorders, Capital Medical University, No. 10 Xitoutiao, You An Men, Beijing 100069, China; yaqi.xu@mail.ccmu.edu.cn (Y.X.); gaoran@mail.ccmu.edu.cn (R.G.); zhangmin@mail.ccmu.edu.cn (M.Z.); zengqi2022@mail.ccmu.edu.cn (Q.Z.); 112020010272@ccmu.edu.cn (G.Z.); xqxxps@mail.ccmu.edu.cn (J.Q.); 2 Laboratory for Clinical Medicine, Capital Medical University, Beijing 100069, China

**Keywords:** systemic lupus erythematosus, autoantibody, Fam210b, single-cell sequencing, erythroid cells

## Abstract

Mitochondrial dysfunction has been increasingly recognized as a trigger for systemic lupus erythematosus (SLE). Recent bioinformatics studies have suggested Fam210b as a significant candidate for the classification and therapeutic targeting of SLE. To experimentally prove the role of Fam210b in SLE, we constructed *Fam210b* knockout (*Fam210b*^−/−^) mice using the CRISPR-Cas9 method. We found that approximately 15.68% of Fam210b^−/−^ mice spontaneously developed lupus-like autoimmunity, which was characterized by skin ulcerations, splenomegaly, and an increase in anti-double-stranded DNA (anti-dsDNA) IgG antibodies and anti-nuclear antibodies(ANA). Single-cell sequencing showed that *Fam210b* was mainly expressed in erythroid cells. Critically, the knockout of *Fam210b* resulted in abnormal erythrocyte differentiation and development in the spleens of mice. Concurrently, the spleens exhibited an increased number of CD71^+^ erythroid cells, along with elevated levels of reactive oxygen species (ROS) in the erythrocytes. The co-culture of CD71^+^ erythroid cells and lymphocytes resulted in lymphocyte activation and promoted dsDNA and IgG production. In summary, *Fam210b* knockout leads to a low probability of lupus-like symptoms in mice through the overproduction of ROS in CD71^+^ erythroid cells. Thus, Fam210b reduction may serve as a novel key marker that triggers the development of SLE.

## 1. Introduction

Systemic lupus erythematosus (SLE) is a chronic autoimmune disease with a global incidence of about 1.5–11/100,000 [1]. It is characterized by abnormal activation of the immune system and high production of autoantibodies [2]. These autoantibodies and antigens form immune complexes that deposit in tissues, causing damage to multiple organs [3]. Genetic factors, environmental influences, and abnormal immune responses to infections often combine to trigger SLE [4]. The pathogenesis of SLE is complex and there is no cure at present.

Mitochondria are emerging as an essential signaling hub in the regulation of both innate and adaptive immunity in eukaryotic cells [5]. Mitochondrial dysfunction is closely related to the occurrence of autoimmune diseases. Mitochondrial DNA (mtDNA) has been found to activate autoreactive T lymphocytes in SLE patients and lead to the production of anti-double-stranded DNA (anti-dsDNA) antibodies [6]. It also can trigger a downstream inflammatory response by activating Toll-like receptors (TLRs), leading to the production of type I interferons (IFNs), which then further destroy immune tolerance [7]. Mitochondrial polymorphism can increase oxidative stress. Oxidative mtDNA accumulate excessively in the mitochondria of neutrophils in SLE patients, and the accumulated oxidative mtDNA is squeezed out through neutrophil extracellular traps (NETs), thus triggering the activation of plasmacytoid dendritic cells (pDCs) and resulting in the production of IFNs [6]. In addition, the production of reactive oxygen species (ROS) caused by mitochondrial dysfunction can interact with proteins, nucleic acids, and lipids to cause oxidative modification of their structures, resulting in membrane phospholipid peroxidation and increased production of active aldehydes [8]. Multiple genome-wide association studies (GWAS) have identified several mitochondrial genes associated with SLE susceptibility [9]. For example, adenosine triphosphate (ATP) synthetase 5/6, immune-related guanosine triphosphatase M protein (IRGM), NADH dehydrogenase (ND) 1, and ND2 have been shown to be involved in mitochondrial biogenesis and mitochondrial autophagy [10]. Targeting cellular metabolic changes in mitochondria has been shown to have therapeutic effects; drugs such as n-acetylcysteine, rapamycin, metformin, Mito Q, and hydroxychloroquine were found to inhibit systemic inflammation in SLE [11]. Hence, the exploration of genes associated with mitochondrial function may hold vital clues to the molecular mechanisms of this disease.

Human and mouse Fam210b is a novel mitochondrial membrane protein. It was first discovered by Aiko Kondo et al. while searching for a GATA1 target gene and it is related to erythrocyte differentiation and development [12]. Fam210b can promote the formation of the mitochondrial iron transport complex by binding with protoporphyrinogen oxidase as a cohesive protein in terminal heme enzyme oligomers, and is a key protein necessary for terminal erythrocyte differentiation and mitochondrial iron input [13]. Moreover, Fam210b can interact with multiple subunits of mitochondrial ATP synthetase, such as α subunits and β subunits, to regulate the differentiation and development of erythrocytes by promoting mitochondrial energy metabolism [14]. Additionally, Fam210b is associated with a variety of tumors, such as those of ovarian cancer, breast cancer, lung cancer, and hepatocellular carcinoma, which can inhibit tumor growth and migration by affecting metabolic reprogramming and the extracellular signal-regulated kinase (ERK)– Protein kinase B (PKB, also known as AKT) protein pathway [15].

Two recent bioinformatics studies have suggested that Fam210b is an important candidate for the classification and therapeutic targeting of SLE [16,17]. To prove the role of Fam210b in SLE via experimental methods, we constructed mice with *Fam210b* knockout and found that *Fam210b* knockout leads to a low probability of lupus-like symptoms in mice. Our study provides evidence for targeting Fam210b as a potential therapeutic and diagnostic strategy for SLE-like autoimmune conditions in the future.

## 2. Results 

### 2.1. Fam210b Knockout Induced Lupus-Like Symptoms in Mice

**Figure 1:**
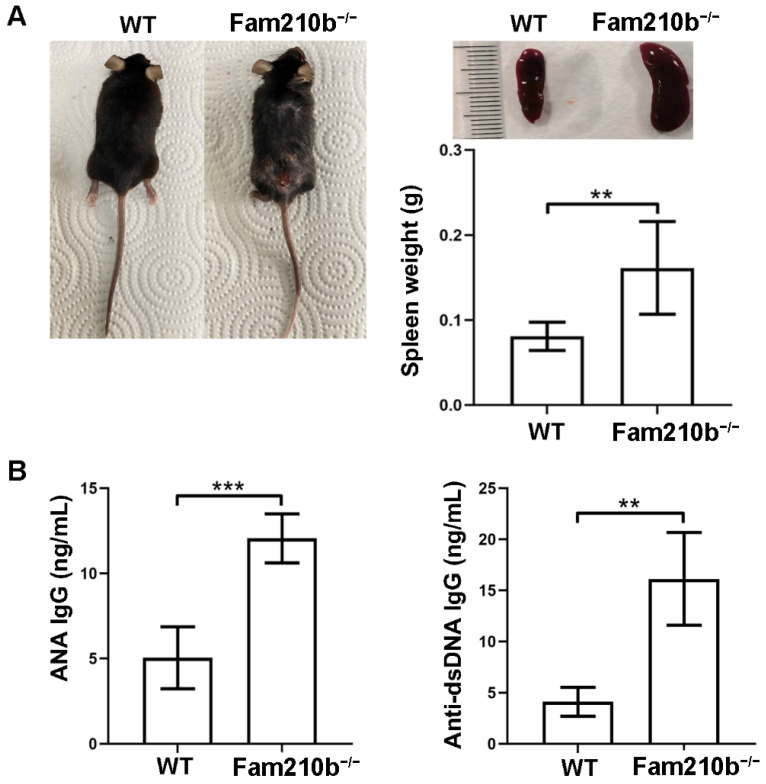
*Fam210b*^−/−^ mice present lupus-like symptoms. Note (**A**) ulceration on the back (**left**), splenic enlargement (**right upper**), and spleen weight (**right lower**) in the diseased mice as taken from 20-week-old *Fam210b*^−/−^ mice. (**B**) ANA (**left**) and anti-dsDNA IgG antibody (**right**) levels in the sera from 20-week-old WT and *Fam210b*^−/−^ mice were determined by ELISA. The data were expressed as mean ± SD, double-tailed *t*-test, ** *p* < 0.01, *** *p* < 0.001.

To investigate the function of Fam210b in SLE, we constructed *Fam210b* knockout (*Fam210b*^−/−^) mice using the CRISPR-Cas9 method. Upon observation, *Fam210b* knockout did not demonstrate any effect on fertility and survival in the mice. Approximately 15.68% of the *Fam210b*^−/−^ mice began to develop skin ulceration and splenomegaly at 12 weeks (Figure 1A). The autoantibody content of the peripheral blood of *Fam210b*^−/−^ mice was analyzed, and it was found that the content of ANA and anti-dsDNA IgG antibodies had increased 2.38-fold (*p* = 0.0009) and 3.91-fold (*p* = 0.0023), respectively (Figure 1B). Due to the specificity of anti-dsDNA antibodies and ANA in the diagnosis of SLE [18], these results suggest that *Fam210b* knockout can induce the development of lupus-like autoimmunity in mice.

**Figure 2:**
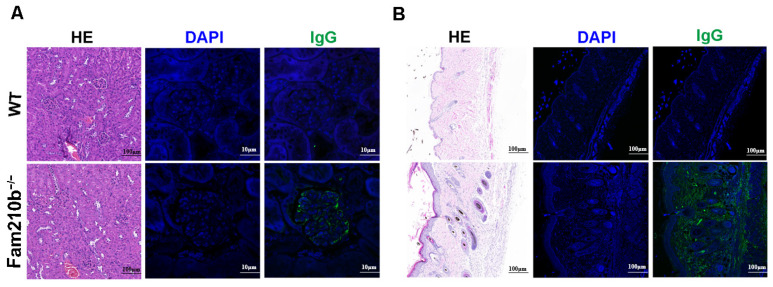
Lymphocyte infiltration and IgG deposition in kidney and skin tissues from *Fam210b*^−/−^ mice. H&E staining and anti-mouse IgG immunofluorescence staining were performed on kidney (**A**) and skin (**B**) cross-sections from 20-week-old WT and diseased *Fam210b*^−/−^ mice. scale bar: 100 μm for H&E staining; 10 μm for immunofluorescence staining.

The production of autoantibodies caused by antigen accumulation plays a central role in the immunopathology of SLE [19]. These autoantibodies can form immune complexes that deposit on the kidneys and skin, causing tissue damage. H&E staining of the kidneys and skin of *Fam210b*^−/−^ mice revealed glomerulonephritis with mesangial hyperplasia and skin lesions with sebaceous hyperplasia and massive lymphocyte infiltration. Immunofluorescence staining revealed IgG deposits in the glomeruli and damaged skin of *Fam210b*^−/−^ mice. These results show that *Fam210b*^−/−^ mice developed glomerulonephritis and skin symptoms (Figure 2). Collectively, our results suggest that *Fam210b*^−/−^ mice can develop lupus-like autoimmunity at a low incidence rate.

### 2.2. Fam210b Is Mainly Expressed in Erythroid Cells

To determine which cell subsets express Fam210b, we performed single-cell sequencing using the spleens from WT and *Fam210b*^−/−^ mice. A total of 16 cell subsets were identified in splenocytes (Supplementary Figure S1A). These cell populations are: CD4^+^ T cells (Cd4T), CD8^+^ T cells (Cd8T), B cells (B), plasma cells, erythroid_prog, erythroid cells, endotheial cells, natural killer cells (NK), lymphomyelod cells, Neutrophils (Neut), pDC, cDC1, Myelod_prog, monocytes, lymphomyelods, red pulp macrophages (RpMacs), and mononuclear macrophages (MoMF). The expression of marker genes in each cell subset is shown in Supplementary Figure S1B.

**Figure 3:**
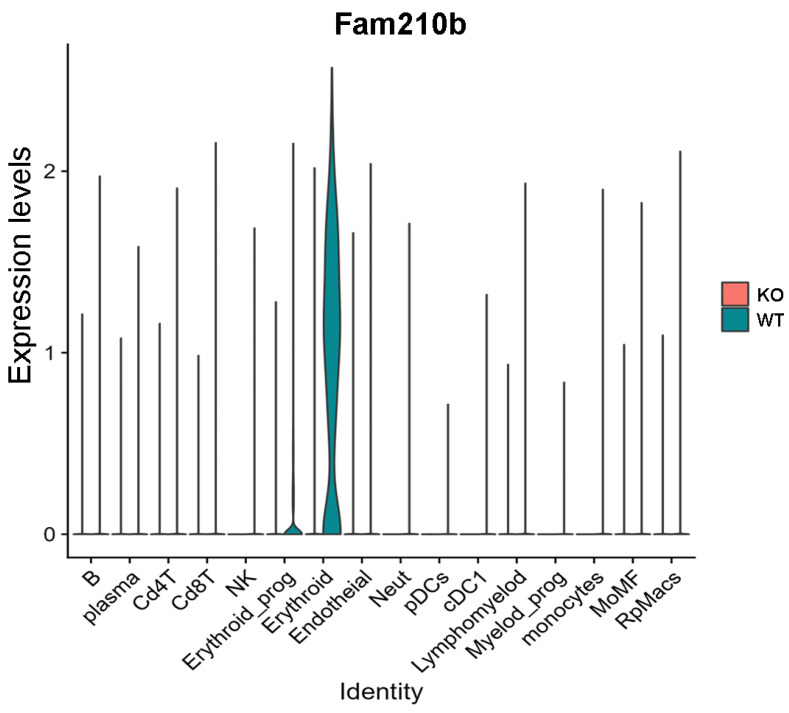
*Fam210b* is mainly expressed in erythrocytes. Splenocytes were collected from 30-week-old WT and *Fam210b*^−/−^ mice and subjected to single-cell sequencing. Cell subsets and their marker gene expressions are identified in Supplementary Figure S1. Violin diagram of *Fam210b* expression in various cell subsets is shown.

The violin map shows that *Fam210b* was mainly expressed in the erythroid cell region, and was less expressed in other cell populations (Figure 3). Additionally, we found that *Fam210b* was completely knocked out in splenic erythroid cells from *Fam210b*^−/−^ mice (Figure 3). Our results indicate that *Fam210b* is mainly expressed in erythroid cells and knockout of *Fam210b* may affect erythrocyte differentiation and development in the spleen.

### 2.3. Fam210b Knockout Inhibited Splenic Erythrocyte Differentiation and Development

**Figure 4:**
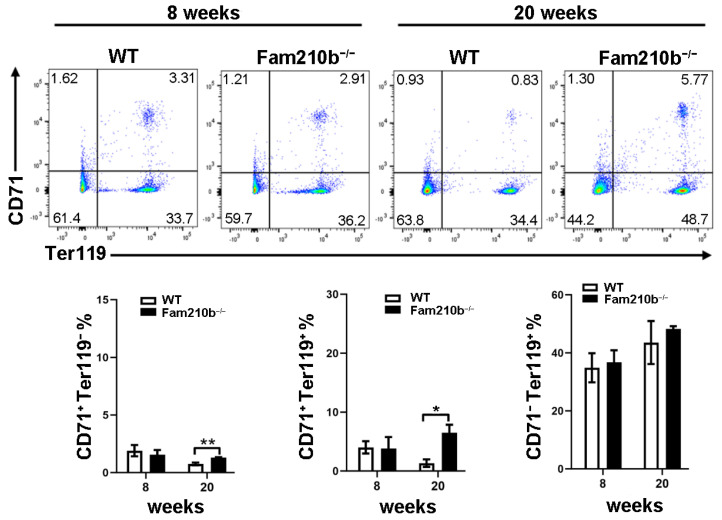
The knockout of *Fam210b* up-regulates CD71^+^ erythrocytes in mice. Splenocytes were collected from 8- and 20-week-old WT and *Fam210b*^−/−^ mice. The percentages of CD71^+^Ter119^−^, CD71^+^Ter119^+^, and CD71^−^Ter119^+^ erythrocytes in splenocytes were determined by flow cytometry. The data were expressed as mean ± SD, two-way ANOVA combined with Bonferroni post hoc test, * *p* < 0.05, ** *p* < 0.01.

To investigate the effect of Fam210b on splenic erythrocyte differentiation and development in mice, splenocytes were extracted from mice at 8 and 20 weeks. The differentiation and development of mouse erythrocytes can be measured by the differential expression of CD71 and Ter119. CD71^+^Ter119^−^ cells mainly contain proerythroblasts and basophilic erythroblasts, while CD71^+^Ter119^+^ cells mainly contain basophilic and polychromatic erythroblasts. CD71^−^Ter119^+^ cells represent mature red blood cells [20]. As the age of the mice increased, we found that the proportion of CD71^+^Ter119^−^ cells and CD71^+^Ter119^+^ cells in the spleen of 20-week mice was significantly reduced compared to 8-week mice (Figure 4). However, the proportion of CD71^+^Ter119^−^ cells and CD71^+^Ter119^+^ cells in the spleen of Fam210b^−/−^ diseased mice was significantly increased than that of the control (*p* = 0.0128) (Figure 4). These results indicated that *Fam210b* knockout inhibited the differentiation and development of erythroid cells, and the proportion of CD71^+^ erythroid cells increased in the spleen of Fam210b^−/−^ diseased mice.

### 2.4. Fam210b^−/−^ Erythroid Cells Stimulate Lymphocyte Activation

**Figure 5:**
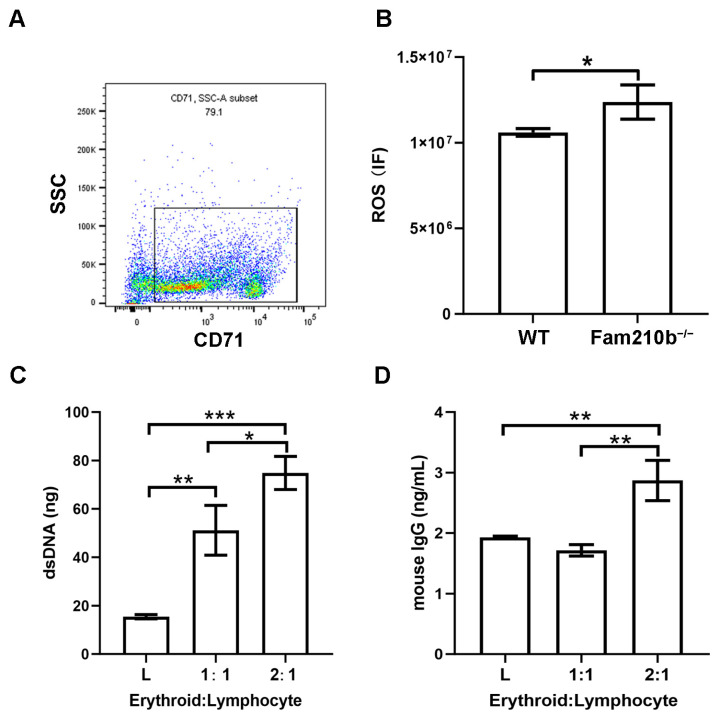
*Fam210b* knockout stimulates the immune response by increasing ROS production. (**A**) The purity of the CD71^+^ erythroid cells was detected by flow cytometry after magnetic bead sorting. (**B**) ROS levels of CD71^+^ erythroid cells in spleens of Fam210b^−/−^ mice. (**C**) dsDNA content in CD71^+^ erythrocyte and splenic lymphocyte co-culture supernatant stimulated by LPS. (**D**) IgG content in the supernatant of CD71^+^ erythrocyte and splenic lymphocyte co-culture stimulated by LPS. The data were expressed as mean ± SD, and the statistical analysis method was a two-tailed unpaired *t*-test, * *p* < 0.05, ** *p* < 0.01, *** *p* < 0.001.

From single-cell sequencing analysis, the molecular functions involved in Fam210b in erythroid cells were shown to have peroxidase activity and oxidoreductase activity. Hence, we performed ROS detection on CD71^+^ erythroid cells in the spleens of Fam210b^−/−^ mice. We first used immunobeads to sort CD71^+^ erythroid cells in the spleen and found that the percentage of CD71^+^ erythroid cells in the sorted cells was 79.1% (Figure 5A). As expected, we found that the ROS content in CD71^+^ erythroid cells from Fam210b^−/−^ mice was significantly higher than that of the control group (*p* = 0.0317) (Figure 5B). Overproduction of ROS can spread outside the cell and activate the immune cell response [11]. It can also interact with proteins, nucleic acids, and lipids to cause oxidative modification of their structures, which can lead to membrane phospholipid peroxidation and increase the production of active aldehydes [8]. Mitochondrial dysfunction and ROS overproduction are commonly present in SLE [21]. To further investigate the immunological function of CD71^+^ erythroid cells, we co-cultured isolated splenic CD71^+^ erythroid cells and lymphocytes. The results showed that dsDNA content and IgG content in the supernatant increased with the increase of the number of Fam210b^−/−^CD71^+^ erythroid cells (Figure 5C,D). These results indicate that Fam210b^−/−^ erythroid cells may lead to the activation of lymphocytes through the overproduction of ROS. The accumulation of CD71^+^ erythroid cells with high ROS levels may trigger the development of SLE.

## 3. Discussion

Our study reveals the unknown function of mitochondrial protein Fam210b in SLE. We demonstrated for the first time that Fam210b deficiency induces lupus-like autoimmunity in mice. 

In previous studies, the function of Fam210b in erythrocyte differentiation and development was only explored in cell lines [14]. In this study, we further identified Fam210b as a key protein in erythrocyte differentiation and development in mice. The absence of Fam210b could lead to the retarding of erythrocyte differentiation and development, resulting in erythrocyte elevation in the spleen. Elevated ROS levels in the erythroblast cells that accumulate in the spleen may be responsible for triggering the development of lupus-like autoimmunity in mice. 

Fam210b knockout induces a low probability of lupus-like symptoms in mice. The occurrence of SLE can be divided into three stages: (1) Autoreactive B-cells escape tolerance through various mechanisms and are activated by helper T-cells, leading to autoantibody production; (2) The accumulation of autoantigens is accompanied by the formation of immune complexes and the activation of myeloid cells, plasmacytoid dendritic cells, and other immune cells; (3) Tissue and organ damage caused by deposition of immune complexes and infiltration of immune cells [22]. In SLE, the BCR and TLR receptors are often dysregulated and lead to the production of autoreactive B-cells [23]. In addition, T–B lymphocyte interaction further enhances the function of B-cells [19]. The continuous interaction between follicular resident T-cells and GC B-cells in GC promotes the survival and proliferation of B-cells through the feedforward cycle process of CD40L, induced T-cell co-stimulatory factors, and B-cell activation factors [24]. The production of autoantibodies resulting from B-cell tolerance disruption, T–B lymphoid interactions, and antigen accumulation play a central role in the immunopathology of SLE [2]. According to recent literature, ANA positivity has gained more priority in SLE classifications, such as those of the American College of Rheumatology (ACR) and the European League against Rheumatism (EULAR) [25]. ANA is composed of anti-nucleosomes, anti-dsDNA, anti-histones, anti-Sm, anti-RNP, anti-RO antibodies, and anti-La antibodies, among which anti-Sm antibodies and anti-dsDNA antibodies are SLE-specific antibodies, and the other antibodies are also found in other autoimmune diseases [22]. ANA is thought to play a role in disease progression through multiple mechanisms, systematically affecting the immune system and local organs such as the brain, kidneys, and skin. The level of anti-dsDNA antibodies is a key autoantibody biomarker of the SLE Disease Activity Index (SLEDAI), some of which can trigger or enhance the inflammatory response and induce tissue deposition [26]. We observed significant elevations in ANA IgG antibody and anti-dsDNA IgG antibody levels. Additionally, we detected renal and skin IgG deposits in the spleens of diseased *Fam210b*^−/−^ mice, collectively indicating that the knockout of Fam210b triggers lupus-like autoimmune responses.

Fam210b is a key protein in promoting erythrocyte differentiation and development. Erythropoiesis, a tightly orchestrated process, involves hematopoietic stem cells (HSCs) generating over 2 × 10^11^ red blood cells daily, primarily in the bone marrow and spleen [27]. Early stages of erythropoiesis include HSCs, megakaryocyte–erythroid progenitor cells (MEPs), burst-forming unit erythroid cells (BFU-Es), and colony-forming unit erythroid cells (CFU-Es), and are regulated mainly by IL-3/IL-3R and stem cell factor (SCF)/c-KIT [28]. During terminal erythropoiesis, proerythroblasts undergo major changes, including cell size reduction as well as nuclear condensation, and begin to produce intensively erythroid lineage-specific proteins, including hemoglobin. Proerythroblasts differentiate into basophilic, polychromatophilic, and orthochromatophilic erythroblasts, successively [29]. This intricate process is rigorously governed by a diverse array of factors, encompassing the activation of hematopoietic cytokines, glucocorticoids, and peroxisome proliferator-activated receptors [30]. Many studies have shown that Fam210b plays an important role in the stage of erythrocyte terminal differentiation [13,14], but its effect on erythrocyte differentiation and development in Fam210b^−/−^ mice has not been studied. To investigate the role of Fam210b in the differentiation and development of mouse erythroid cells, we used the differential expression of CD71 and Ter119 to measure terminal erythropoiesis. Our study showed that *Fam210b* knockout led to the blockage of the differentiation and development of erythroid cells in mice. Although published GWAS studies did not reveal any extant association between *Fam210b* mutations and hematologic traits [31,32], the study of Fam210b in mice has shown that it is a key protein in promoting erythrocyte differentiation and development. These results also suggested that some patients with Fam210b-related anemia need to take preventative measures against developing SLE.

There was an accumulation of CD71^+^ erythroid cells in the spleens of the diseased *Fam210b*^−/−^ mice and an excessive production of ROS. CD71^+^ erythroid cells are immature red blood cells, including erythroblasts and reticulocytes, which contain mitochondria [33]. Compared with mature red blood cells, the ROS level in CD71^+^ erythroid cells is higher [34], which plays an important role in promoting the process of enucleation of red blood cells and decreases as red blood cells mature [35]. Extensive research has demonstrated that CD71^+^ erythroid cells exhibit a crucial immunosuppressive function in neonates, tumors, and anemia. Notably, these cells are physiologically abundant in the spleen of newborn mice [36] and humans [37]. In newborn mice, the abundance of CD71^+^ erythroid cells inhibited the immune response of T-cells to infection through the expression of arginase-2, effectively mitigating excessive inflammatory responses when faced with infection [36]. Furthermore, ROS generated by CD71^+^ erythroid cells exerts a regulatory effect on the immune response within the tumor microenvironment, specifically modulating T-cell activation, apoptosis, and hyporeactivity [38]. The co-culture of *Fam210b*^−/−^ CD71^+^ erythroid cells and lymphocytes in vitro resulted in the production of dsDNA and IgG, suggesting that Fam210b^−/−^ CD71^+^ erythroid cells may promote immune response by releasing ROS.

Most ROS in cells are produced by the mitochondrial respiratory complex, and ROS can act as a trigger for mitochondria, leading to the activation of mitochondrial permeability transition pores, changes in mitochondrial autophagy, and changes in mitochondrial dynamics [39]. These mitochondrial mechanisms triggered by ROS can cause mtDNA to escape under stress, acting as damage-associated molecular patterns (DAMPs), by binding to pattern recognition receptors (PRRs) on the surface of immune cells and triggering innate immune responses [40]. The release of large amounts of mtDNA may be a source of autoantigens by activating autoreactive B-cells, leading to the production of autoantibodies [41]. In addition, there are many types of ROS, among which superoxide anion (O_2_^•−^), hydrogen peroxide (H_2_O_2_), and hydroxyl radical are important subtypes that cause cell damage [38]. Studies have shown that H_2_O_2_ has a long half-life and is one of the few active oxygen molecules that can freely diffuse through cell membranes [42]. After activation of NADPH oxidase, H_2_O_2_ is first produced in the extracellular space and diffuses into the cell through the plasma membrane as a secondary messenger [43]. In the immune system, activation of lymphocytes usually requires the formation of close contact synapses between two cells. This synapse forms between antigen-presenting cells and T-cells, as well as between B-cells [44]. As lymphocyte activation is more oxidative, H_2_O_2_ is more stable outside the cell than inside the cell [44]. When cells with high H_2_O_2_ levels interact with B-cells, the dose response specifically activated by the B-cell antigen is transferred to the lower antigen concentration [42]. Therefore, even B-cells with low affinity BCRs can be stimulated to produce antibodies when exposed to highly active H_2_O_2_ [42]. Our results suggest that *Fam210b* knockout leads to the accumulation of CD71^+^ erythroid cells with high ROS levels. *Fam210b* knockout may lead to overproduction of ROS by affecting mitochondrial dysfunction, and high levels of ROS may activate the immune response by spreading to immune cells, which eventually leads to the occurrence of lupus-like autoimmunity.

There are many limitations to our study. First, we found that *Fam210b* knockout resulted in a low probability of a lupus-like phenotype. Further analysis is needed to determine why most mice did not develop lupus-like autoimmunity and the extent of the association between reduced *Fam210b* expression and incidence. Then, the fact that *Fam210b* knockout led to an increase in ROS in CD71+ erythroid cells indicates that FAM210b may be a key molecule in regulating ROS metabolism, and the mechanism through which Fam210b affects ROS metabolism needs further investigation. In addition, to investigate the effect of *Fam210b*^−/−^ CD71^+^ erythroid cells on immune cells, we only isolated spleen erythroid cells and co-cultured them with lymphocytes. The results showed that dsDNA and IgG content were increased with the proportion of erythroid cells, suggesting that Fam210b may activate B-cells by releasing ROS. However, the mechanism leading to the increase of dsDNA content and B-cell activation needs to be further explored. The spleen is known largest filter of RBCs in the body where the smallest openings for RBC passage are located [45]. The parenchyma of the spleen consists of white myeloid nodules and sheathing, containing mainly T and B lymphocytes, which are scattered in the red pulp, a spongy tissue that makes up 75% of the volume of the spleen. The red pulp includes the splenic sinuses, which are blood vessels alongside the connective tissue of the splenic cord. About 10% of the blood entering the spleen enters so-called open circulation, where red blood cells are forced from the umbilical cord into the venous sinuses [45]. We have only observed the stimulating effect of erythroblasts on lymphocytes through isolation in vitro, but the interaction mechanism between erythroblasts and lymphocytes in spleen tissue needs further investigation in *Fam210b*^−/−^ mice.

In conclusion, deletion of Fam210b is associated with the development of lupus-like symptoms, suggesting that Fam210b holds significant potential as a key molecular trigger for the development of autoimmunity. Our research offers compelling evidence that can aid in the diagnosis and treatment of SLE. In the future, it is necessary to further explore the mechanism of Fam210b in SLE and pay more attention to mitochondrial homeostasis in SLE.

## 4. Materials and Methods

### 4.1. Mice

The animal study protocol was approved by the Institutional Animal Care and Use Committee at Capital Medical University (Approval code: AEEI-2020-187. Approval date: 1 December 2020). *Fam210b*^−/−^ mice were constructed using CRISPR-Cas9 technology in the C57BL/6N mouse strain by Cyagen Biosciences Inc., Suzhou, Jiangsu Province, China. By designing a pair of gRNA sequences to identify the exon sequence of the target gene, Cas9 protease was guided to effectively cut the DNA double strand to form a double strand break, and the repair after the damage results in the knockout of *Fam210b*. After breeding screening, homozygous *Fam210b*^−/−^ mice were obtained. Age- and gender-matched wild-type (WT) C57BL/6N mice in the same litter were used as the control group.

### 4.2. Extraction of Peripheral Blood and Spleens from Mice

After anesthesia, peripheral blood was collected from mice. The collected peripheral blood was placed on the table for about 3 h, then the serum was collected by centrifuge and stored at −80 °C after subpackaging. The spleens of the mice were dissected, washed with PBS, and weighed. The spleen was placed on a 70 μm cell sieve and triturated with 1640 medium or PBS (Thermo Fisher Scientific Inc., Waltham, MA, USA) to make a single-cell suspension. Splenocytes were collected for subsequent analysis.

### 4.3. Single-Cell Sequencing of Spleens

Splenocytes from two 30-week-old diseased *Fam210b*^−/−^ and two 30-week-old WT mice were used for single-cell sequencing at Beijing Genomics institution, Beijing, China. The original data analysis after sequencing was carried out using R package “Seurat 4.0” (New York Genome Center, New York, NY, USA). We integrated data through a standard pre-processing workflow, including QC and selecting cells for further analysis, mitochondrial gene elimination, normalizing the data, identification of highly variable features, performance of linear dimensional reduction, determining the ‘dimensionality’ of the dataset, clustering the cells, and running a non-linear dimensional reduction (UMAP/tSNE). The cell types were then defined based on genetic markers and differential gene expression (DEG). The “cellratio” function was used to count the proportion of cell populations, and the “subset” function was used to extract and reanalyze cell subsets. DEG was calculated using the Wilcoxon rank-sum test and Bonferroni correction for the adjusted *p*-values. According to the “Seurat” official tutorial, specific gene expression levels were analyzed based on the “RNA” counting slot display by drawing single gene maps and violin maps, and a DEG-based GO enrichment analysis network was created using functional annotation bioinformatics microarray analysis (DAVID). Finally, we used the ggplot2 and heatmap packages in the R package to visualize the annotation results.

### 4.4. Flow Cytometry

All cell experiments were strictly prepared on ice. Cells (1 × 10^6^ cells/sample) were washed with fluorescence-activated cell sorting staining buffer (phosphate-buffered saline, 2% foetal bovine serum or 1% bovine serum albumin, 0.1% sodium azide). All samples were incubated with fluorescence-conjugated anti-mouse CD71, and Ter119 (Thermo Fisher Scientific Inc., Waltham, MA, USA) was used to analyze erythroid cell subsets. Cells were analyzed on a LSRFortessa flow cytometer (BD Biosciences Inc., San Jose, CA, USA). The data were analyzed using FlowJo (version: 10, Tree Star Inc., Ashland, OR, USA).

### 4.5. ELISA Assay

Anti-dsDNA IgG antibody and ANA analyses were performed with the ELISA kit (CUSABIO Biotechnology Inc., Wuhan, China). Briefly, the standard and sample were added to a coated plate and incubated at 37 °C for 2 h. Next, biotin-labeled dsDNA or ANA was added to each well, incubated at 37 °C for 2 h, and washed with washing solution three times. Then, HRP-labeled working solution was added and incubated at 37 °C for 1 h. After five washes, chromogenic solution was added and the absorbance was measured at 450 nm with a microplate reader (Molecular Devices Inc., ShangHai, China). 

### 4.6. H&E Staining and Immunofluorescence

Mouse kidneys and skin from diseased *Fam210b*^−/−^ mice were fixed by paraformaldehyde, of which the concentration was 4%, and then embedded in paraffin. Sliced sections of 5 μm thickness were affixed into HistoBond^®^ adhesive microscopic slides. These slides were stained with hematoxylin and eosin (H&E) and then stored at room temperature. For the immunofluorescence assay, slides were incubated at 70 °C for one hour to dissolve the wax. Then, dewaxing and rehydration was carried out by a Leica ST5010 Autostainer XL (Leica Biosystems Inc., Deerfield, IL, USA). After sodium citrate antigen repair, the slides were incubated in blocking buffer (5% FBS in PBST) for 1 h at room temperature. Sections were incubated with Alexa Fluor™ 488 anti-Mouse IgG (Thermo Fisher Scientific Inc., Waltham, MA, USA) at 4 °C overnight. Confocal fluorescence microscopy (Leica Microsystems Inc., Deerfield, IL, USA) was used to analyze and evaluate IgG deposits in the kidneys and skin of mice.

### 4.7. CD71^+^ Erythroid Cell Sorting

Splenocytes were filtered to obtain a single-cell suspension. Then, 10^8^ cells were re-suspended in 500 µL MACS buffer with 10 µL FITC-conjugated anti-mouse CD71 antibody (Thermo Fisher Scientific Inc., Waltham, MA, USA) and incubated at 4–8 °C for 30 min. After, 10 mL PBS buffer was added to wash the cells. Each 10^7^ cells were added with 90 µL MACS buffer and 10 µL FITC magnetic beads (Miltenyi Biotec Inc., Bergisch Gladbach, Germany) and incubated at 4–8 °C for 15 min. After washing with PBS, cells were re-suspended with 500 uL MACS buffer and added to the LS column (Miltenyi Biotec Inc., Bergisch Gladbach, Germany). After three washes, the column was removed from the separator and placed in a suitable collection tube. An appropriate amount of IMDM (Thermo Fisher Scientific Inc., Waltham, MA, USA) was added to the LS column and immediately flushed out fractionally with the magnetically labeled cells by firmly applying the plunger.

### 4.8. Co-Culture and Stimulation

In order to investigate the immunological function of Fam210b^−/−^ CD71^+^ erythroid cells, CD71^+^ erythrocytes from the spleens of diseased mice were sorted using magnetic beads. Splenic lymphocytes from WT mice were isolated using mouse lymphocyte separator (Solarbio Corporation, Beijing, China), and then co-cultured in 6-well plates according to the ratios of erythrocytes:lymphocytes: 2:1, 1:1, and 0:1. The next day, the cells were stimulated with 10 µg/mL LPS (L2630 from *Escherichia coli* 0111:B4; Sigma-Aldrich, St. Louis, MO, USA). After 3 days of stimulation, the supernatant and cells were collected.

### 4.9. dsDNA Content Detection

The content of dsDNA in cell culture medium was detected with the EZQuant dsDNA quantitative kit (abcam Inc., Boston, MA, USA) by combining the dsDNA Dye with dsDNA and sheltering it from light for 5 min after the reaction. The fluorescence intensity was measured at Ex/Em = 480 nm/530 nm by a microplate reader.

### 4.10. ROS Content Detection

The ROS content in CD71^+^ erythroid cells was detected using the Reactive Oxygen Species Assay Kit (Meilunbio Biotechnology Inc., Shanghai, China). Firstly, the reactive oxygen species probe (DCFH-DA) was diluted with serum-free medium at 1:1000, and the final concentration was 10 μM. Then, the cells were suspended in DCFH-DA working liquid and incubated in a cell culture incubator at 37 °C for 20–30 min. The cells were washed three times with serum-free medium to fully remove the DCFH-DA that did not enter the cells. The fluorescence intensity was measured at Ex/Em = 488 nm/525 nm with the microplate reader.

### 4.11. Statistical Analysis

All experimental data were statistically analyzed and mapped using GraphPad Prim software (version: 10; GraphPad Software Inc., San Diego, CA, USA). Comparisons between the two groups were made using a two-tailed *t*-test. Multi-group comparisons were made using one-way ANOVA combined with Dunnett’s post hoc test. The multivariate comparison was conducted using two-way ANOVA combined with Bonferroni post hoc test. All data are expressed as mean ± SD. A *p* < 0.05 was considered statistically significant.

## Data Availability

Dataset available on request from the authors. The raw data supporting the conclusions of this article will be made available by the authors on request.

## Supplementary Materials

The following supporting information can be downloaded at: https://www.mdpi.com/article/10.3390/ijms25137253/s1.

## List of Abbreviations

SLE	systemic lupus erythematosus
mtDNA	mitochondrial DNA
anti-dsDNA	anti-double-stranded DNA
LPS	lipopolysaccharide
ANA	anti-nuclear antibody
TLRs	toll-like receptors
IFNs	type I interferons
NETs	neutrophil extracellular traps
pDCs	plasmacytoid dendritic cells
ROS	reactive oxygen species
GWAS	genome-wide association studies
ATP	adenosine triphosphate
IRGM	immune-related guanosine triphosphatase M protein
ND	NADH dehydrogenase
Fam210b^−/−^	Fam210b knockout
ERK	extracellular signal-regulated kinase
ELISA	enzyme-linked immunosorbent assay
