# Supplementary material for: Deletion of the Mitochondrial Membrane Protein Fam210b Is Associated with the Development of Systemic Lupus Erythematosus
Source: Int J Mol Sci. 2024 Jul 1;25(13):7253. doi: 10.3390/ijms25137253 (PMC11241391; doi:10.3390/ijms25137253)
Supplement: Supplementary file 1 [file ijms-25-07253-s001.zip › ijms-3070700-supplementary.pdf]

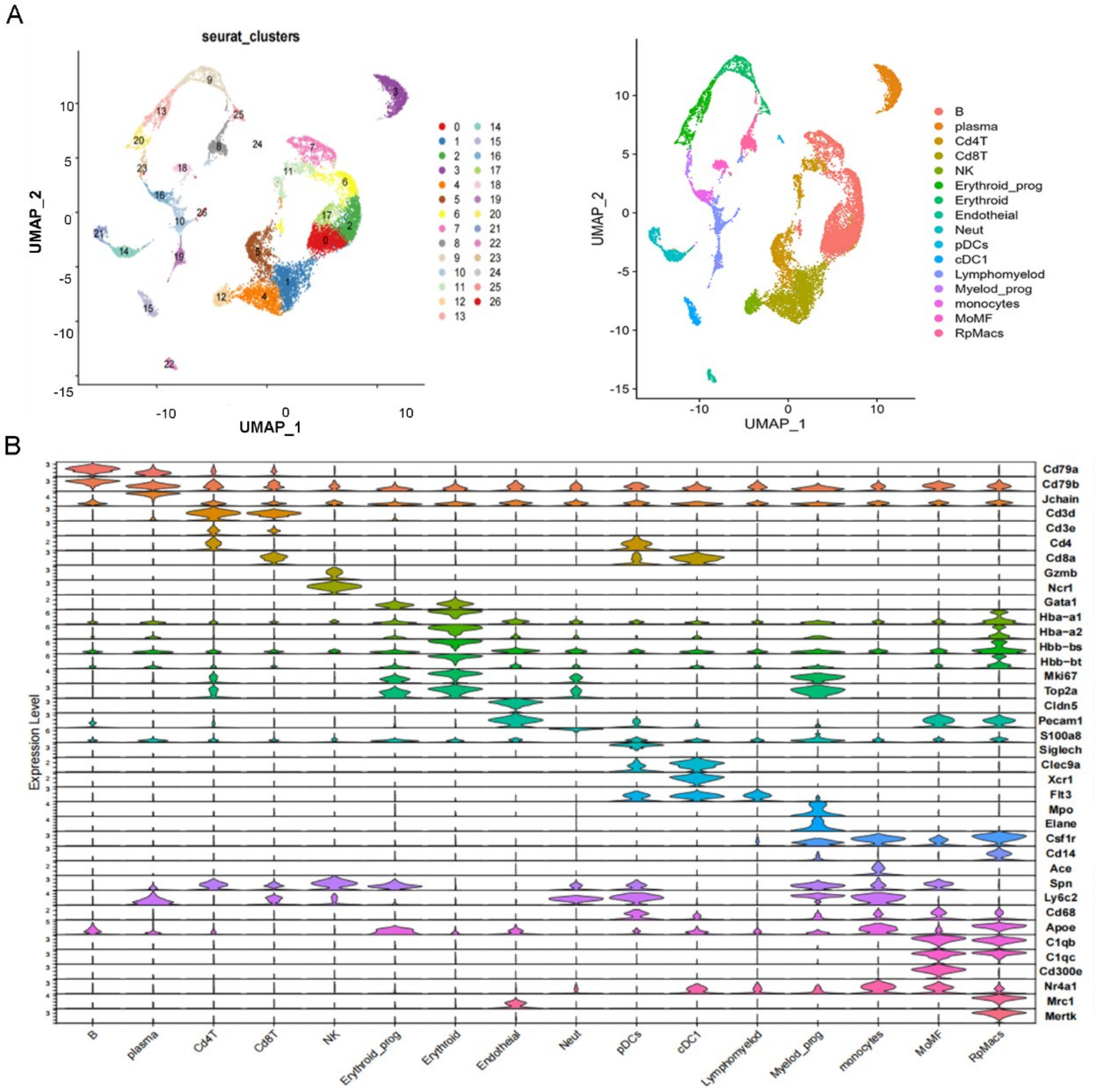

Supplementary Figure S1. Cell population in spleen of Fam210b$^{-/-}$ mice were analyzed by single cell sequencing. Splenocytes were collected from 30-week-old WT and Fam210b$^{-/-}$ mice and subjected to single cell sequencing. (**A**) UMAP of cell subsets with dimensionality reduction after single cell sequencing. (**B**) Violin map of selected marker genes to define 16 major cellular clusters.
